# Outcomes used to measure the clinical application of neonatal palliative and/or end-of-life care in neonatal settings: a systematic review

**DOI:** 10.1136/archdischild-2024-328252

**Published:** 2025-01-31

**Authors:** Katie Gallagher, Kathy Chant, Veronica Parisi, Mehali Patel, Helena Dunbar, Fauzia Paize, Sophie Bertaud, Agnes Agyepong, Alexandra Mancini, Myra Bluebond-Langner, Neil Marlow

**Affiliations:** 1 University College London, London, UK; 2 Sands, London, UK; 3 Together for Short Lives, Bristol, UK; 4 Liverpool Women’s NHS Foundation Trust, Liverpool, UK; 5 University of Oxford, Oxford, UK; 6 Great Ormond Street Hospital, London; 7 Global Black Maternal Health, London, UK; 8 Chelsea and Westminster Hospitals NHS Foundation Trust, London, UK

**Keywords:** Palliative Care, Intensive Care Units, Neonatal, Neonatology, Health services research

## Abstract

**Objectives:**

Standardised reporting of outcomes in neonatal palliative and/or end-of-life care would facilitate comparison of practice and lead to more informed decisions about practice. We systematically reviewed evidence evaluating outcomes currently used to characterise the clinical provision of palliative and/or end-of-life care in neonatal settings.

**Methods:**

A systematic review following Preferred Reporting Items for Systematic Reviews and Meta-Analyses guidelines was undertaken using Ovid Medline, Ovid Embase, OVID PsycINFO, OVID MIDIRIS and EBSCOhost CINAHL. No date or language restrictions were used. Studies were included if they measured or reported outcomes related to the clinical practice of neonatal palliative care in a neonatal unit.

**Results:**

Of 7998 records identified through database searching, 20 articles were included. Identified studies were retrospective chart reviews. No studies used standardised outcomes and all used proxy outcome measures. Results were organised according to the WHO domains of paediatric palliative care. All studies (n=20) reported documentation of physical symptoms and functional status (physical domain); six documented parental emotional and support needs (psychological domain); four reported sibling support and wider family presence (social and cultural domain), and three reported support from spiritual services (spiritual domain).

**Conclusion:**

Despite neonatal death accounting for the largest category of child death under 5 years of age, there are no standardised outcomes from which to characterise or develop clinical practice. Developing a core outcome set for neonatal palliative and end-of-life care would ensure that services can be compared using reliably collected and collated data and help advance care in this area.

**WHAT IS ALREADY KNOWN ON THIS TOPIC**

**WHAT THIS STUDY ADDS**

- The first review of outcomes used to measure the clinical provision of neonatal palliative and/or end-of-life care in neonatal settings.
- Insight into the documentation of neonatal palliative care in neonatal settings globally.
- An understanding of the limitations of the current evidence regarding the provision of palliative care in neonatal settings.

**HOW THIS STUDY MIGHT AFFECT RESEARCH, PRACTICE OR POLICY**

- There is limited evidence from which to develop meaningful outcome measures to effectively evaluate the efficacy of neonatal palliative care.
- The role of documentation in neonatal palliative care requires clarification.
- The development of standardised outcome measures for neonatal palliative care is needed to meet the needs of all families and healthcare professionals.

## Background

 In neonatal practice, there is wide variation in the care provided for families whose infants have chronic illnesses or are facing death or uncertain outcomes. This is reflected in differences in support available to mitigate the anxiety and distress faced by families, potentially impacting their short- and long-term mental health and well-being.^1 2^ Equally, there is little evidence to guide healthcare professionals in managing symptoms that infants may display at the end of their lives. In turn, this leads to disparate end-of-life care practices, which are often based on experience rather than evidence.^1^ Standardising outcomes can enhance patient care by allowing the results of multiple studies to be compared, leading to better-informed decisions.^3^,^5^ These can then be combined into a core outcome set to be reported in future palliative care studies.

The WHO defines neonatal palliative care as the active approach to improving the quality of life of infants and their families when facing health-related suffering associated with life-threatening or chronic illness, including end-of-life care planning and preparation.^6 7^ There is a growing awareness among parent support groups and healthcare professionals of the need for studies of the efficacy of different palliative care strategies to support families with infants who die in neonatal units or survive with complex needs.^8^ Standardised reporting of outcomes would facilitate direct comparison of the core aspects of different neonatal palliative care and/or end-of-life care strategies, including symptom identification and management.^5 9^ This would facilitate a high-quality evidence base to form the basis for local and national policy and its evaluation. This systematic review forms the first stage of the development of a core outcome set for neonatal palliative and/or end-of-life care (herein neonatal palliative care) in the UK (the NeoPACE study).^10^

In this systematic review, our aim was to identify outcomes used to characterise the clinical practice of palliative care in neonatal settings. Secondary objectives were to identify formal measurement tools used to evaluate these outcomes and assess the methodological quality of the studies included.

## Methods

The study was conducted in accordance with Preferred Reporting Items for Systematic Reviews and Meta-Analyses guidelines.^11^ The search terms and strategy were developed with a subject-specific librarian (VP) (online supplemental table 1) and applied in the following databases: Ovid Medline, Ovid Embase, OvidPsycINFO, Ovid MIDIRIS and EBSCOhost CINAHL. Forward citation tracking was also undertaken to identify additional relevant studies. Studies were included if they determined or reported outcomes related to the clinical practice of neonatal palliative care in a neonatal unit or if they explored neonatal palliative care as part of a broader study and the neonatal data had been analysed and presented separately. Studies were excluded if they reported the impact of palliative care educational interventions on healthcare staff knowledge and attitudes, reported hypothetical scenarios or focused on delivery room death (due to the unique challenges of providing palliative care in this setting). No date limits were set as neonatal care remains a relatively new specialist area of healthcare. No language exclusion criteria were applied if a full translation was available/feasible using Google Translate. Quality assessment of eligible studies was planned using the relevant tool for the methodological approach, such as the Critical Appraisal Skills Programme (CASP).^12^ The databases were last searched on 7 August 2024.

The core outcome set study is registered with the Core Outcome Measures in Effectiveness Trials initiative (https://www.comet-initiative.org/Studies/Details/1470) and the systematic review with the International Prospective Register of Systematic Reviews (PROSPERO) (CRD42023451068).^5 13^ No changes were made to the study protocol throughout the review. Search results were imported into EndNote and de-duplicated. De-duplicated results were uploaded into Rayyan, a web-based systematic review tool, for screening and to facilitate data analysis.^14^ All papers were screened by title and abstract and then full text. Two researchers (KG and KC) independently reviewed all results. Independent data extraction was facilitated by Microsoft Excel collating information on the study overview (author, title, year, journal and country), methods (aim, methodology, eligibility criteria, participants, intervention and PPI involvement), outcomes (timing and definitions) and any associated outcome measurement tools (names and definitions). Results were then unblinded on Rayyan to facilitate analysis. Following discussion, there were no discrepancies, which required further moderation by a third team member.

We systematically reviewed all outcomes and any definitions and measurement instruments. To facilitate analysis, reported outcomes were categorised according to the WHO domains of paediatric palliative care, including physical, psychological, sociocultural and spiritual.^7^ The two researchers independently undertook data synthesis within each domain using content analysis across all findings to ensure that similar data was grouped together for comparison. Where there were discrepancies in what was reported within each domain (such as information around the use of pain medications or reporting of decision-making), all aspects of the data were extracted and compared, looking for similarities or differences between papers.

## Results

**Figure 1:**
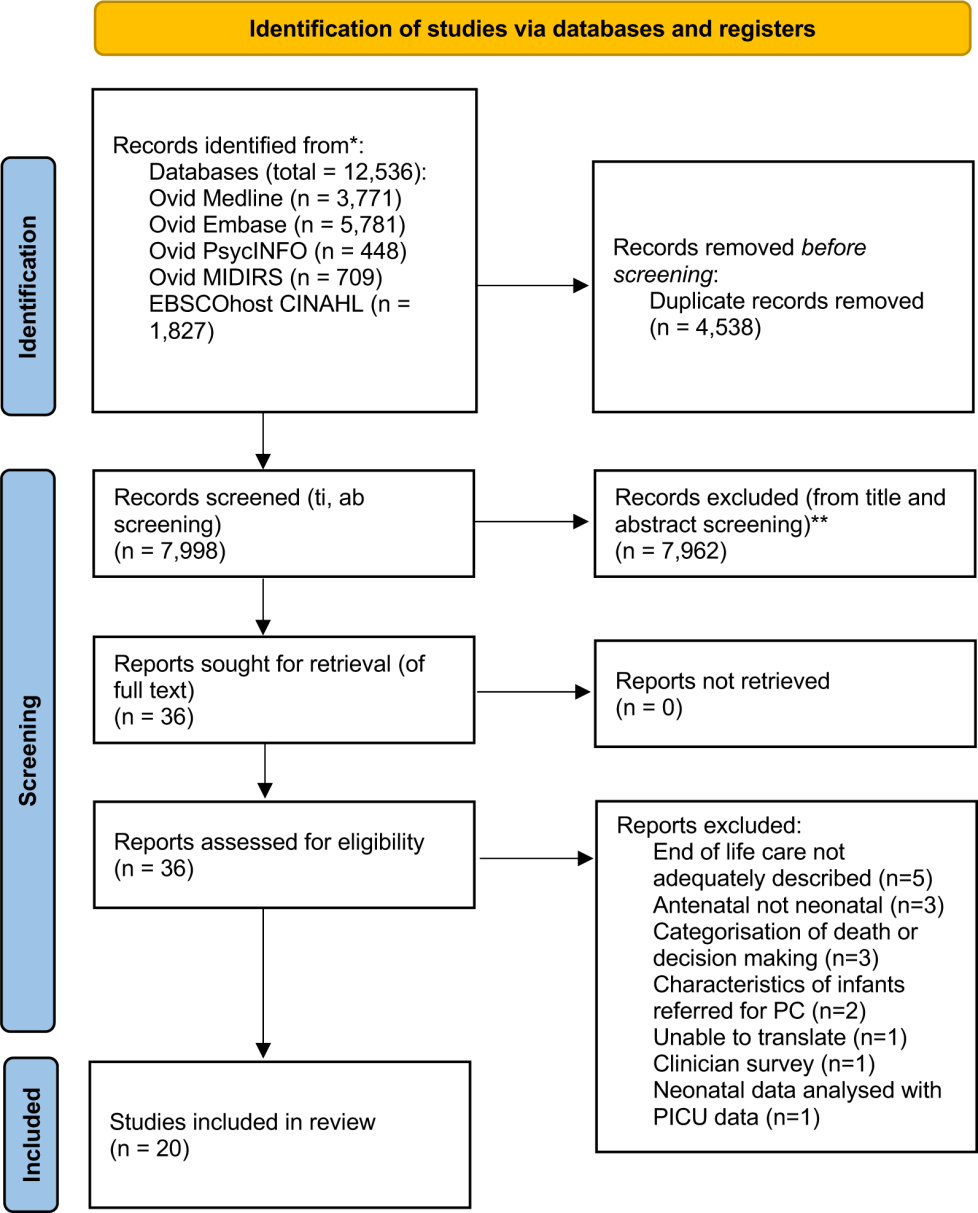
Preferred Reporting Items for Systematic Reviews and Meta-Analyses flow diagram of study identification and inclusion for the systematic review of the clinical application of neonatal palliative and/or end-of-life care.

Following automatic de-duplication, the search strategy identified 7998 papers. The titles and abstracts of all 7998 were screened for potential inclusion. We identified 36 papers for full-text review, and 20 subsequently met the inclusion criteria for the systematic review (figure 1).^115^,^33^

### Quality assessment

The quality assessment of the papers is limited as we identified no randomised controlled trials or prospective observational studies exploring symptom management or interventions during neonatal palliative care; hence, we present a narrative review of the results. All studies used retrospective chart reviews for which there is no formal methodological assessment tool. Indeed, most studies did not identify a specific reporting tool used for the review (n=13, 65%); four (20%) created their own, and three (15%) adopted an existing tool. Only three studies (n=3, 15%) discussed the rigour of their methods and/or any piloting of data collection. Of the 10 studies that discussed data extraction, eight reported the researchers involved and the resolving of discrepancies in data collection; however, no information was given regarding interrater reliability of information extracted from the clinical record.

None of the 20 studies used standardised outcome measures to evaluate neonatal palliative care (online supplemental table 2). All studies used proxy outcomes to describe symptoms, symptom management and interventions during palliative and/or end-of-life care in the neonatal unit, as defined by neonatal professionals. All 20 studies used retrospective chart reviews. Nine of the 20 studies were from the USA, four from Europe (n=2 Spain, n=1 from Germany and Portugal, respectively), two from Latin America and one paper from Southeast Asia (n=1 Taiwan), Canada, Australia and New Zealand, respectively. One study compared practices in the USA, Canada and the Netherlands. Publication dates ranged from 1997 to 2024. The majority of studies were published before 2017 (n=13, 65%); only seven (35%) studies were published between 2021 and 2024. Four papers (20%) provided a definition of neonatal palliative care. Eight studies (40%) presented demographic data on infant ethnicity.

### Domains of neonatal palliative care

#### Physical

All 20 papers reported on aspects of physical symptoms and functional status.^115^,^33^ The management of neonatal pain through the use of opioids and/or sedatives was most commonly reported (n=15). Variation was found in the reporting of opioid analgesics during the end-of-life phase (definitions ranged from 48 to 72 hours before death), ranging from 74% (n=94/127) in a single-centre 10-year review to 91% (n=30/33) in a study reporting the care of 33 infants in a tertiary neonatal unit in the USA.^16 29^ Only three (15%) studies reported the dosage of opioid analgesics administered during the infant’s terminal care.^1 24 31^ The largest study to explore analgesic exposure during the end-of-life phase was that of Zimmerman *et al*,^33^ whose team analysed the notes of 19 726 infants from 348 neonatal units in the USA between 1997 and 2012.^33^ They identified that only 27% of 5366 infants received an opioid analgesic on their day of death, in contrast to 48% who received either an analgesic or sedative at some point during their hospitalisation.

There was minimal information on how pain was measured in practice; only one study identified the pain scale (Neonatal Pain, Agitation and Sedation Scale and Face, Legs, Activity, Cry and Consolability scale) used to assess infant pain during their end-of-life phase.^20^

10 studies (50%) aimed to explore documentation around aspects of decision-making for infants with palliative and/or end-of-life care needs.^1518 19 21 24 27^,^30 32^ A definition of what decision-making entailed was not provided in any of the studies. Lack of definition resulted in variation in the documentation of the number and nature of decision-making discussions held and/or such matters as the rationale behind the withdrawal of treatment documented (eg, ‘quality of life’ or ‘poor prognosis’). In a single-centre study from New Zealand, for 83% of infants (n=120/145), a discussion to ‘withdraw treatment’ from the baby had been documented.^29^ In a similar single-centre study in Australia, a ‘redirection of care’ conversation had been documented in 93% of cases.^24^ In contrast, other studies sought documentation of the rationale behind decision-making. In a study of eight neonatal units across five Latin American countries, the rationale was documented in only 40% (n=40) of the notes of all neonatal deaths,^17^ similar to 47% of included infants in a single-centre study in Spain.^30^

Only one study evaluated documented parental participation in decision-making in two neonatal units between 1997 and 1998, 15/18 families were identified as participating in the decision to remove ventilatory support from their critically unwell baby.^15^

Five studies identified medical interventions or treatment intensity preceding infant death, but there was variation between studies in what was classified as an intervention (eg, resuscitation, blood sampling and palliative surgery) or the time base over which these were recorded (from 1 to 7 days before death).^17 20 23 28 32^ Eight studies recorded the time from decision-making and/or ventilator withdrawal until death, which varied from 15 min to 22 days.^15 19 21 25 27 29 31 32^

Four studies reported on the documentation of symptoms exhibited by infants during the end-of-life phase.^16 20 24 32^ The most common symptom reported across all studies was respiratory difficulties, which was identified in 24%–96.8% of infants across the studies.^16 20 24 32^ Further symptoms documented in infants around the time of death included (but were not limited to) pain, seizures and agitation.

Two studies (11%) reported the use of non-pharmacological measures to keep infants comfortable during end-of-life care including (among others) positioning, containment care and kangaroo care.^20 32^ One further study was unable to find sufficient documentation in the care records examined.^1^

One study reported on the documentation of support for 11% (n=7) of mothers who wished to breastfeed their dying baby.^30^ One study reported on the documentation of lactation advice after bereavement, which was provided to 77% of women.^24^

#### Psychological

Documentation of the emotional and support needs of families was identified in eight studies through documentation of meetings with or referrals to specialist services, among which included palliative care teams, social workers, chaplaincy, psychologists, psychiatrists and child life teams.^15 16 18 20 24 25 28 29^

Eight studies reported on either parental presence or parental holding of their baby around the time of the baby’s death. Of these, four studies reported on the documentation of parental presence when the baby died, ranging from 3.7% to 98.5%.^17 21 28 30^ Two studies reported parental presence when the baby died in 49.9% and 86% of cases, respectively, additionally reporting that ‘most’ parents were holding their baby at this time.^15 32^ One study reported that 89.8% of parents held their baby following the withdrawal of treatment, and one study reported that 72% of parents held their baby as they died.^25 27^ In these final two studies, it was reported that a member of staff held the baby as they died if parents were unable to do so.^25 27^

Six studies reported on the documentation of creating memories with families, or ‘memory making’, prior to the death of the baby, including handprints, photographs and personalised albums.^15 24 25 29 30 32^

No studies reported the documentation of the implications of organisational or financial issues for families during end-of-life care, such as accommodation, car parking fees or food vouchers. Only one study documented support for siblings of the index child.^24^ One study evaluated the ongoing palliative care needs of infants who survived following discharge from the neonatal unit,^18^ identifying a median of eight different specialisms required to support the infants and families at both discharge and at 1 year of age.

#### Spiritual/religious

10 studies reported on the documentation of spiritual or religious services provided to parents around the time of the infant’s death, including support from chaplaincy, the offer of a religious ceremony or ritual, cultural services and baptism of the baby.^16^,^1821 24 25 27^

## Discussion

In this narrative review, we aimed to identify reported outcomes following neonatal palliative care. Despite a large literature, we were only able to report proxy outcomes from 20 studies, which were all retrospective chart reviews and thus provided no evidence of the efficacy of intervention strategies in this area. The reporting of care offered to infants and families varied widely and, indeed, seven studies concluded that there was little documentation during the final hours of a baby’s life to understand what support was made available for families included in their populations. It seems clear that while clinical trials or large observational studies may not historically have been undertaken due to ethical or economic reasons in neonatal palliative care, there is a need for prospective standardised data collection in this area to allow for the effects of different interventions to be evaluated and to set standards for good practice. Stakeholder engagement (including parents, parent representatives, healthcare professionals and researchers) is central to this process to ensure that the research reflects the needs of families and healthcare professionals.

The study has several limitations. There is potential geographical and temporal bias, as many studies were conducted in the USA and the majority before 2017. This may not reflect current practice or advancements in neonatal care. Whereas documentation of care is required in many countries, the lack of standardised data collection also means that we cannot be certain if other care was provided but not (or poorly) recorded. Contributing studies provided little information about data extraction, and researchers limited their reports to what they considered to be the focus of their investigation, hence the variable nature of information included in the included reports. Reliance on proxy outcome measures, rather than direct and validated measures, could lead to inconsistencies in how outcomes are reported and interpreted. This review therefore reflects what was recorded in a variety of settings and not necessarily what was provided. This first study to draw reports together in this area highlights a lack of standardisation for reporting events during neonatal palliative care.

The variability among studies included in this review reflects a lack of clarity about how palliative care is integrated into neonatal settings, compounded by the allusive language used to describe palliative care practice: for example, ‘withdrawal of treatment’, ‘comfort care’, ‘redirection of care’ and ‘parallel planning’ are phrases used within and between studies. The healthcare team relies on clearly articulated documentation as part of interprofessional communication.^34^ The importance of clarity and inclusiveness in records is vital to ensure that families receive high quality and consistent patient care.

The identification of symptoms and signs that are managed using a range of care strategies forms a key part of neonatal palliative care.^7 35^ How these are managed is important for the child’s well-being during the palliative care process and is particularly important from the parent’s perspective, as parents who recall their baby having more symptoms at the end of life may perceive that their infant suffered more.^36^ Despite the central importance of this area in paediatric oncology, for example, where patients often experience a multitude of symptoms in their end-of-life phase (including shortness of breath, pain, nausea and vomiting, and restlessness),^37^ there are few descriptions of the characteristics of the symptoms encountered in neonatal palliative care. Among management strategies, there is also wide variation in the prescription and dosing of opioid analgesics, and there are no commonly reported pain or comfort scales with which to evaluate the outcomes of such treatment.

Within several settings, offering parents the opportunity to stay with and hold their infant at the end of their infant’s life is an important aspect of care. Reports suggest some variation in this as recorded in care records as described above. This is a complex measure, which has not been clarified in reports. Parents may feel unable or not wish to be present at the time of their baby’s death for a range of reasons. Some of this may be cultural, which is unclear from the data reported, or the timing of deterioration may make this impossible. In these situations, staff may substitute for parents in holding the infant, which generally should be done with express permission.

The provision of support facilities and services for parents and staff is likewise important but is frequently not indicated in the care record. Parental support from their family, community and spiritual advisers may be a key source of comfort but often is not recorded clearly. Support for staff is also important, as neonatal staff are at an increased risk of burnout when working in this emotionally challenging environment.^38^

Healthcare interventions are recorded more frequently than social support, perhaps because there is some continuing routine in doing so during palliative care. However, there is a great need to understand the needs of different ethnic groups (in whom neonatal death may be more common), families with different degrees of social advantage/disadvantage and different spiritual situations where community leaders may provide an important support structure during and after neonatal death.^39 40^

## Conclusion

Deaths during neonatal care comprise the majority of mortality during the first 5 years. It is important to ensure that neonatal palliative care services are well developed and effective. One of the first steps in this process may be to establish a core outcome set for palliative and end-of-life care to ensure that services and strategies can be compared using reliably recorded and collated data. This could facilitate a prospective approach to palliative and end-of-life care research, which could provide insights into crucial aspects such as symptoms infants experience at the end of life and thus appropriate pain management tools to assess these, leading to improved infant care. This first review in the area has highlighted that current descriptive reports are solely based on chart reviews without standardised definitions or data collection. It highlights the lack of an evidence base from which effective neonatal palliative and end-of-life care can be developed, which would be enhanced by the development of a core outcome set.

## Supplementary material

10.1136/archdischild-2024-328252 online supplemental file 1

10.1136/archdischild-2024-328252 online supplemental file 2

## Data Availability

All data relevant to the study are included in the article or uploaded as supplementary information.
