# Supplementary material for: Outcomes used to measure the clinical application of neonatal palliative and/or end-of-life care in neonatal settings: a systematic review
Source: Arch Dis Child Fetal Neonatal Ed. 2025 Jan 31;110(5):e328252. doi: 10.1136/archdischild-2024-328252 (PMC12418539; doi:10.1136/archdischild-2024-328252)
Supplement: online supplemental file 1 [file fetalneonatal-110-5-s001.pdf]

**Database: Ovid MEDLINE(R) ALL<1946 to August 07, 2024**	**Embase <1974 to 2024**	**Maternity & Infant Care Database (MIDIRS) <1971 to August 2024**	**APA PsycInfo <1806 to August Week 2 2024**	**CINAHL**
**1** Palliative Care/<br>**2** Advance Care Planning/<br>**3** palliative care.tw.<br>**4** (palliative adj2 nursing).tw.<br>**5** advance* care plan*.tw.<br>**6** bereavement care.tw.<br>**7** end of life.tw.<br>**8** comfort care.tw.<br>**9** or/1-8<br>**10** exp Infant, Newborn/<br>**11** Perinatal Care/<br>**12** Infant Death/<br>**13** Perinatal Death/<br>**14** (neonat* or neo-nat*).tw.<br>**15** perinatal.tw.<br>**16** (newborn* or new-born*).tw.<br>**17** infant*.tw.<br>**18** or/10-17<br>**19** 9 and 18	**1** palliative therapy/<br>**2** advance care planning/<br>**3** palliative care.tw.<br>**4** (palliative adj2 nursing).tw.<br>**5** advance* care plan*.tw.<br>**6** bereavement care.tw.<br>**7** end of life.tw.<br>**8** comfort care.tw.<br>**9** or/1-8<br>**10** perinatal care/<br>**11** newborn death/<br>**12** perinatal death/<br>**13** newborn/<br>**14** (neonat* or neo-nat*).tw.<br>**15** perinatal.tw.<br>**16** (newborn* or new-born*).tw.<br>**17** infant*.tw.<br>**18** or/10-17<br>**19** 9 and 18	**1** palliative care.tw.<br>**2** (palliative adj2 nursing).tw.<br>**3** advance* care plan*.tw.<br>**4** bereavement care.tw.<br>**5** end of life.tw.<br>**6** comfort care.tw.<br>**7** or/1-6<br>**8** (neonat* or neo-nat*).tw.<br>**9** perinatal.tw.<br>**10** (newborn* or new-born*).tw.<br>**11** infant*.tw.<br>**12** or/8-11<br>**13** 7 and 12	**1** palliative care/<br>**2** palliative care.tw.<br>**3** (palliative adj2 nursing).tw.<br>**4** advance* care plan*.tw.<br>**5** bereavement care.tw.<br>**6** end of life.tw.<br>**7** comfort care.tw.<br>**8** or/1-7<br>**9** perinatal period/<br>**10** neonatal period/<br>**11** (neonat* or neo-nat*).tw.<br>**12** perinatal.tw.<br>**13** (newborn* or new-born*).tw.<br>**14** infant*.tw.<br>**15** or/9-14<br>**16** 8 and 15	**S1** MH Palliative Care<br>**S2** MH Advance Care Planning<br>**S3** MH Perinatal Care<br>**S4** MH "Perinatal Death") OR (MH "Infant Death")<br>**S5** (MH "Infant, Newborn+")<br>**S6** TI "palliative care" OR AB "palliative care"<br>**S7** TI (palliative N2 nursing) OR AB (palliative N2 nursing)<br>**S8** TI "advance* care plan*" OR AB "advance* care plan*"<br>**S9** TI "bereavement care" OR AB "bereavementcare"<br>**S10** TI "end of life" OR AB"end of life"<br>**S11** TI "comfort care" OR AB "comfort care"<br>**S12** TI (neonat* or "neo- nat*") OR AB (neonat* or "neo-nat*")<br>**S13**TI perinatal OR ABperinatal<br>**S14** TI (newborn* or "new-born*") OR AB (newborn* or "new-born*")<br>**S15** TI infant* OR AB infant*<br>**S16** S1 OR S2 OR S6 OR S7 OR S8 OR S9 OR S10 OR S11<br>**S17** S3 OR S4 OR S5 OR S12 OR S13 OR S14 OR S15<br>**S18** S16 AND S17

Supplementary Information S1: Search terms and strategy to systematically review the clinical application of neonatal palliative and/or end-of-life care
