# Supplementary material for: Outcomes used to measure the clinical application of neonatal palliative and/or end-of-life care in neonatal settings: a systematic review
Source: Arch Dis Child Fetal Neonatal Ed. 2025 Jan 31;110(5):e328252. doi: 10.1136/archdischild-2024-328252 (PMC12418539; doi:10.1136/archdischild-2024-328252)
Supplement: online supplemental file 2 [file fetalneonatal-110-5-s002.pdf]

| Study | Journal (Country) | Title | Aim | Methods | Number of infants | Outcomes measured | WHO domains of palliative care |
|---|---|---|---|---|---|---|---|
| Abe et al 2001 | MCN. The American journal of maternal child nursing | End of life in the NICU. A study of ventilator withdrawal<br><br>(USA) | To determine what is being documented about the EoL process for newborns who die following removal of life support. | Retrospective chart analysis (2NNUs) (1997-1998) | 18 infants | 1.orders for & process of ventilator removal<br>2. medications neonate received at the end of life<br>3. decision making<br>4. family support | Physical (1,2,3)<br>Psychological (4) |
| Carter et al 2004 | Pediatrics | Circumstances surrounding the deaths of hospitalized children: opportunities for pediatric palliative care (USA) | To describe documentation of care during EoL for infants and children. | Retrospective chat review (paediatric hospital: 1 NNU) (2001) | 33 neonates (105 in total, incl. paediatric) | 1.clinical management (e.g. communication, DM, ventilator removal)<br>2.family and child interdisciplinary support (social / spiritual/psychological support)<br>3. pain and symptom management (pain medication at end of life) | Physical (1,3)<br>Psychological / social / spiritual (2) |
| Currie et al 2023 | Journal of Pain and Symptom Management | Patterns of Pediatric Palliative and End-of-Life Care in Neonatal Intensive Care Patients in the Southern U.S (USA) | To describe NPC patterns & treatment intensity during last 48hrs of life in infants receiving NPC consultation | Retrospective chart review of 2 NNUs (2009-2017) | 195 infants | 1. patterns of NPC (timing of NPC consultation, hospice referral, DNRs spiritual care),<br>2. treatment intensity in last 48hrs prior to death (i.e. CRP, intubation / ventilation, line placement surgery) | Physical (2)<br>Psychological/ spiritual (1) |

| | | | | | | | |
|---|---|---|---|---|---|---|---|
| Deming et al 2022 | Journal of Pain and Symptom Manageme nt | Care Intensity and Palliative Care in Chronically Critically Ill Infants (USA) | To describe documentatio n of care during EoL for infants and children. | Retrospectiv e chart review (3 NNUs) (2016-2019) | 273 infants | 1.care intensity at discharge (types & number of technology/ medication/specialist input required)<br>2.social work / spiritual / child life involvement,<br>3. Follow up @1yr: types & number of technology/ medication/specialist input required, readmissions | Physical (1,3)<br>Psychological / spiritual / social (2) |
| Fajardo et al 2012 | Acta Paediatrica | End of life, death and dying in neonatal intensive care units in Latin America (Latin America) | To determine how decisions and processes regarding dying infants take place in units in Hispanic Latin America | Retrospectiv e chart review (8 NNUs) (2009) | 100 infants | 1.clinical management (e.g. DNR, CPR)<br>2.pain management<br>3.decision making | Physical (1,2,3) |
| Fortney et al 2015 | Advances in Neonatal Care | Medical record documentation and symptom management at the end of life in the NICU (USA) | To study symptoms exhibited by neonates at the EoL and treatments used to manage these as documented in medical records | Retrospectiv e chart review (1 NNU) (2007-2010) | 20 infants | 1.prenatal history<br>2.on-going therapy during EOL<br>3.pain and comfort interventions used<br>4.signs and symptoms exhibited during EOL<br>5.parental presence at time of death | Physical (1,2,3,4)<br>Psychological (5) |
| Fortney et al 2023 | Journal of Palliative Medicine | Characteristics of Critically Ill Infants at the | To identify the characteristic | Retrospectiv e chart review | 476 infants | 1.signs and symptoms exhibited during EoL<br>2.medications and comfort measures | Physical (1,2,3,4) |

| | | | | | | | |
|---|---|---|---|---|---|---|---|
| | | End of Life in the Neonatal Intensive Care Unit (USA) | s of infants who died over a 10yr period (treatment, symptoms) | (1 NNU) (2009-2019) | | 3.interventions during EoL (e.g. surgery, suction, TPN)<br>4.EoL characteristics (e.g. referral to specialist PC team, hospice, DNR, CPR) | |
| Garcia-Alix et al 2013 | Acta Paediatrica | Neonatal hypoxic-ischaemic encephalopathy: most deaths followed end-of-life decisions within three days of birth (Spain) | To determine circumstances surrounding EoL of infants with HIE over 10yr period | Retrospective chart review (1 NNU) (2000-2009) | 70 infants | 1.clinical management (decision making, CPR, time to death)<br>2.complementary studies involved in decision making (e.g. EEG)<br>3.use of sedation during EoL<br>4.parental psychological/spiritual support<br>5.parental presence at time of death | Physical (1,2,3)<br>Psychological/spiritual (4,5) |
| Garten et al 2011 | European Journal of Pain | End-of-life opioid administration on neonatal and pediatric intensive care units: Nurses' attitudes and practice (Germany) | To determine how nurses at 2 NICUs and 3 PICUs view indication for opioids of hypothetical NICU patient, and whether attitudes in accordance with current practice of opioid administration at that hospital. | Observational study and retrospective chart review (2 NNUs) (2008-2009) | 103 infants | 1.pain and sedative use during EoL<br>2.use of pain assessment scales | Physical (1,2) |

| | | | | | | | |
|---|---|---|---|---|---|---|---|
| Gibelli et al 2021 | Journal of Pediatrics and Child Health | Limits of therapeutic intervention in a tertiary neonatal intensive care unit in patients with major congenital anomalies in Brazil (Latin America) | To describe the care provided at EoL for newborns admitted with congenital anomalies to NNU to determine (a)DM and (b)treatment provided in 48hrs before death | Retrospective chart review (1 NNU) (2015-2018) | 74 infants | 1.decision making<br>2.clinical management (ventilatory support, length of stay, IV access)<br>3.medication exposure (antibiotics/sedation/analgesia.inotropes and/or prostoglandin)<br>4.invasive procedures offered (e.g. dialysis, medications) | Physical (1,2,3,4) |
| Gilmore et al 2017 | Journal of Pediatrics and Child Health | Adequacy of palliative care in a single tertiary neonatal unit (Australia) | To characterise EoL care provision in NNU by assessing performance with components of NPC | Retrospective chart review (1 NNU) (2012-2014) | 46 infants | 1.Indicators of neonatal palliative care: communication, resuscitation planning, preferred location of death, symptoms during EOL, symptom management plan (including use of opiates during EOL), MDT approach, caring for carers, memory making, spiritual care, specialised bereavement care | Physical<br>Psychological<br>Spiritual |
| Groden et al 2024 | Journal of Perinatology | End of life care in a level IV outborn neonatal intensive care unit (USA) | To describe care surrounding the end of life (EOL) in the neonatal intensive care unit (NICU). | Retrospective chart review (1 NNU) 2017-2021 | 208 infants | 1.Clinical information (e.g. use of assisted conception, antenatal history, clinical management)<br>2.Social information (e.g. chaplaincy visit, specialist PC consultation)<br>3.Parental health information (e.g. mental health diagnosis, religious identify, language) | Physical (1)<br>Psychological/social (2,3,4) |

| | | | | | | | |
|---|---|---|---|---|---|---|---|
| | | | | | | 4.parental presence at time of death | |
| Janvier et al 2011 | Journal of Pediatrics | Whom are we comforting? An analysis of comfort medications delivered to dying neonates (USA, Netherlands, Canada) | To clarify use of EOL comfort medication in different NNUs | Retrospective chart review (4 NNUs) (2005-2006) | 151 infants | 1. DNR orders<br>2.comfort medication during EOL (analgesics, sedatives, NMBs)<br>3.comfort medication (analgesia and/or sedative) pre/post extubation (if ventilator withdrawn) | Physical (1,2,3,4,5) |
| Lam et al 2016 | Palliative Medicine | A descriptive report of end-of-life care practices occurring in two neonatal intensive care units (Canada) | To explore neonatal deaths and EoL practices in 2 tertiary NNUs | Retrospective chart review (2 NNUs) (2009-2013) | 227 infants | 1.decision making<br>2.use of comfort medication (analgesic and sedatives) during EOL<br>3.use of medical technologies during EOL<br>4.location of EOL<br>5.parental presence at time of death<br>6.time to death | Physical (1,2,3,6)<br>Psychological (1,4,5) |
| Moura et al 2011 | Clinics | End of life in the neonatal intensive care unit (Portugal) | To evaluate the care given to neonates and their families in terminal situations and the changes that have occurred one decade later. | Retrospective chart review (1 NNU) (1992-1995 & 2002-2005) | Total: 313 infants | 1. therapeutic activities during EOL (e.g. ventilation, antibiotics, TPN)<br>2.use of pain scoring tool and administration of opioids or sedatives<br>3.family support (psychological support)<br>4. decision-making<br>5. parental presence at time of death | Physical (1,2,4)<br>Psychological (3,5) |

| | | | | | | | |
|---|---|---|---|---|---|---|---|
| Ng et al 2022 | BMJ Supportive and Palliative Care | Palliative care in a tertiary neonatal intensive care unit: a 10-year review (New Zealand) | To review how care goals and palliative care pathway implemented and documented | Retrospective chart review (1 NNU) (2006-2015) | 145 infants | 1. decision making<br>2.Comfort care (any intervention used to ease symptoms or distress)<br>3. psychosocial support (e.g. memory making, spiritual support)<br>4.time taken from DM to PC implementation<br>5.staff support (time taken from death to debrief) | Physical (1,2,4) Psychological / spiritual (3,5) |
| Palomo et al 2023 | Jornal de Pediatria | Implementing palliative care, based on family centred care, in a highly complex neonatal unit (Spain) | To describe the main causes and modes of death of patients admitted for neonatal care | Retrospective chart review (1 NNU) (2009-2019) | 344 infant deaths during study period. 256 died following transition to PC and included in study. | 1.cause of death<br>2.decision making (and transition to palliative care)<br>3.acceptance of autopsy<br>4.use of medication during EoL<br>5.place of death<br>6.memory making<br>7.referrals to palliative care unit | Physical (1,2,3,4,5) Psychological/spiritiau (6) Social (7) |
| Partridge et al 1997 | Pediatrics | Analgesia for dying infants whose life support is withdrawn or withheld (USA) | To determine frequency of opioid administration during WiLST | Retrospective chart review (1 NNU) (1989-1992) | 120 infants | 1.decision making<br>2.opiod administration and dosage during EOL<br>3. time to death following life support discontinuation | Physical (1,2,3) |
| Peng et al 2012 | Journal of Critical Care | To explore the conditions of dying infants in NICU in Taiwan (Taiwan) | To document EoL care (final 7 days life), signs /symptoms of infants and | Retrospective chart review (2002-2008) | 61 infants | 1.decision making (including DNRs)<br>2.symptoms exhibited during EOL<br>3.symptom management (e.g. surgery, suctioning, medication)<br>4.parent support (e.g. breastfeeding during EOL, memory making) | Physical (1,2,3) Psychological/ social (4,5) |

| | | | | | | | |
|---|---|---|---|---|---|---|---|
| | | | interventions offered to parents | | | 5.parental presence at time of death | |
| Zimmerman et al (2015) | Journal of Pediatrics | Sedatives and Analgesics Given to Infants in Neonatal Intensive Care Units at the End of Life (USA) | To describe the administration of sedatives and analgesics at the end of life in a large cohort of infants in North American neonatal intensive care units | Retrospective chart review (348 NNUs) (1997-2012) | 19,726 infants | 1.exposure to sedatives and/or analgesics during (a) hospitalisation (b) day of death | Physical (1) |

Supplementary Table 2: characteristics and outcomes measured in the included studies exploring outcomes used to measure the clinical application of neonatal palliative and/or end-of-life care in neonatal settings
